# Fibrates are an essential part of modern anti-dyslipidemic arsenal: spotlight on atherogenic dyslipidemia and residual risk reduction

**DOI:** 10.1186/1475-2840-11-125

**Published:** 2012-10-11

**Authors:** Alexander Tenenbaum, Enrique Z Fisman

**Affiliations:** 1 Cardiac Rehabilitation Institute, Sheba Medical Center, Tel-Hashomer, 52621, Israel; 2 Sackler Faculty of Medicine, Tel-Aviv University, Tel-Aviv 69978, Israel; 3 Cardiovascular Diabetology Research Foundation, Holon 58484, Israel

**Keywords:** Atherogenic dyslipidemia, Bezafibrate, Combined fibrate/statin therapy, Fenofibrate, Metabolic syndrome, Residual cardiovascular risk, Type 2 diabetes

## Abstract

Currently the world faces epidemic of several closely related conditions: obesity, metabolic syndrome and type 2 diabetes (T2DM). The lipid profile of these patients and those with metabolic syndrome is characterized by the concurrent presence of qualitative as well as quantitative lipoprotein abnormalities: low levels of HDL, increased triglycerides, and prevalence of LDL particles that are smaller and denser than normal. This lipid phenotype has been defined as *atherogenic dyslipidemia*. Overwhelming evidences demonstrate that all components of the atherogenic dyslipidemia are important risk-factors for cardiovascular diseases. Optimal reduction of cardiovascular risk through comprehensive management of atherogenic dyslipidemias basically depends of the presence of efficacious lipid-modulating agents (beyond statin-based reduction of LDL-C). The most important class of medications which can be effectively used nowadays to combat atherogenic dyslipidemias is the fibrates. From a clinical point of view, in all available 5 randomized control trials beneficial effects of major fibrates (gemfibrozil, fenofibrate, bezafibrate) were clearly demonstrated and were highly significant in patients with atherogenic dyslipidemia. In these circumstances, the main determinant of the overall results of the trial is mainly dependent of the number of the included appropriate patients with atherogenic dyslipidemia. In a meta-analysis of dyslipidemic subgroups totaling 4726 patients a significant 35% relative risk reduction in cardiovascular events was observed compared with a non significant 6% reduction in those without dyslipidemia. However, different fibrates may have a somewhat different spectrum of effects. Currently only fenofibrate was investigated and proved to be effective in reducing microvascular complications of diabetes. Bezafibrate reduced the severity of intermittent claudication. Cardinal differences between bezafibrate and other fibrates are related to the effects on glucose metabolism and insulin resistance. Bezafibrate is the only clinically available pan - (alpha, beta, gamma) PPAR balanced activator. Bezafibrate decreases blood glucose level, HbA1C, insulin resistance and reduces the incidence of T2DM compared to placebo or other fibrates. Among major fibrates, bezafibrate appears to have the strongest and fenofibrate the weakest effect on HDL-C. Current therapeutic use of statins as monotherapy is still leaving many patients with atherogenic dyslipidemia at high risk for coronary events because even intensive statin therapy does not eliminate the residual cardiovascular risk associated with low HDL and/or high triglycerides. As compared with statin monotherapy (effective mainly on LDL-C levels and plaque stabilization), the association of a statin with a fibrate will also have a major impact on triglycerides, HDL and LDL particle size. Moreover, in the specific case of bezafibrate one could expect neutralizing of the adverse pro-diabetic effect of statins. Though muscle pain and myositis is an issue in statin/fibrate treatment, adverse interaction appears to occur to a significantly greater extent when gemfibrozil is administered. However, bezafibrate and fenofibrate seems to be safer and better tolerated. Combined fibrate/statin therapy is more effective in achieving a comprehensive lipid control and may lead to additional cardiovascular risk reduction, as could be suggested for fenofibrate following ACCORD Lipid study subgroup analysis and for bezafibrate on the basis of one small randomized study and multiple observational data. Therefore, in appropriate patients with atherogenic dyslipidemia fibrates- either as monotherapy or combined with statins – are consistently associated with reduced risk of cardiovascular events. Fibrates currently constitute an indispensable part of the modern anti-dyslipidemic arsenal for patients with atherogenic dyslipidemia.

## Atherogenic dyslipidemia

Currently the world faces epidemic of closely related conditions: obesity, metabolic syndrome and type 2 diabetes (T2DM) [1-6]. A strong correlation between T2DM and cardiovascular diseases is well established [7-9]. Also for the metabolic syndrome (MetS) the best available evidence from randomized control trials (RCT) and large meta-analyses systematically had shown increased risk of cardiovascular events [10-14]. The recent and largest meta-analysis [12] included near one million patients (total n = 951,083). The investigators concluded that the MetS is associated with a 2-fold increase in cardiovascular outcomes and a 1.5-fold increase in all-cause mortality rates.

**Figure 1:**
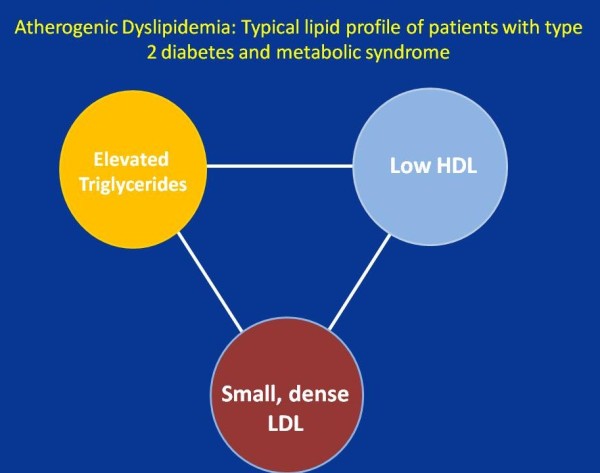
Atherogenic Dyslipidemia triad: the lipid profile which is typical for patients with type 2 diabetes and the metabolic syndrome is characterized by the low HDL-C, increased triglycerides and prevalence of small, dense LDL particles.

The lipid profile of patients with T2DM and MetS is characterized by the concurrent presence of qualitative as well as quantitative lipoprotein abnormalities: low levels of high density lipoprotein cholesterol (HDL-C) (<50 mg/dl in women, < 40 mg/dl in men), increased triglycerides (TG >150 mg/dl), and prevalence of low density lipoprotein (LDL) particles that are smaller and denser than normal (Figure 1). This lipid phenotype has been defined as *atherogenic dyslipidemia*[15-20]. Interestingly, elevated LDL cholesterol (LDL-C) level is not typical of T2DM nor MetS. Overwhelming evidences demonstrate that all components of the atherogenic dyslipidemia are important risk-factors for cardiovascular diseases [21-25].

Particularly, a strong association exists between elevated triglycerides and cardiovascular disease. However, the extent to which triglycerides directly promote disease or represent a biomarker of risk has been debated for decades. The largest and most comprehensive recent meta-analysis included 29 prospective studies and 262,525 participants, proving a strong and highly significant association between triglycerides and coronary risk. Adjustment for HDL-C attenuated the magnitude but did not abolish the significant association between triglycerides and coronary risk [26]. The triglyceride -rich environment has been shown to be strongly associated with an atherogenic lipoprotein phenotype or atherogenic dyslipidemia [22]. In the United States, the National Health and Nutrition Examination Survey (NHANES) has monitored biomarkers of cardiovascular risk for 3 decades. Accordingly, increases in fasting serum triglyceride levels were observed between surveys conducted in 1976–1980 and 1999–2002 [27]. Also, nonfasting triglyceride strongly correlated with coronary risk [28,29]. There is a broad agreement that reverse cholesterol transport, the process of transporting excess cholesterol from the arterial wall’s foam macrophages to the liver, bile, and feces is one of HDL’s important anti-atherogenic properties. Circulating HDL particles are greatly heterogeneous with a very complex metabolic profile. HDL-C measures the cholesterol content of nascent HDL, HDL2, and HDL3 particles and is, therefore, a crude marker of reverse cholesterol transport, whereas non-HDL-cholesterol is a valid marker of coronary risk [30-34].

Optimal reduction of cardiovascular risk through a comprehensive management of atherogenic dyslipidemias basically depends of the presence of efficacious lipid-modulating agents (beyond statin-based reduction of LDL-C). However, most of these agents are currently under serious concerns: niacin after negative AIM HIGH study [35] and before HPS-2 THRIVE trial results, and cholesteryl ester transfer protein (CETP) -inhibitors and glitazars are still in controversial developments and not available for clinical use. Omega-3 polyunsaturated fatty acids supplementation seems to not really influence major cardiovascular outcomes [36]. Therefore, the single class of medications which can be at the moment effectively used to combat atherogenic dyslipidemia beyond statins is only fibric acid derivatives - fibrates.

## The role of fibrates in the management of atherogenic dyslipidemia

Fibrates are used in clinical practice for about half century due to their ability to substantially decrease triglyceride levels and increase HDL. All fibrates are peroxisome proliferators-activated receptors (PPARs) α agonists. Fibrates enhance the oxidation of fatty acids (FA) in liver and muscle and reduce the rate of hepatic lipogenesis, thereby reducing secretion of very-low-density lipoprotein (VLDL) triglycerides. The increased uptake of triglyceride-derived fatty acids in muscle cells results from an increase in lipoprotein lipase (LPL) activity in adjacent capillaries and a decrease in the apolipoprotein CIII (Apo CIII) concentration mediated transcriptionally by PPAR alpha. The decrease in apolipoprotein CIII reduces the inhibition of LPL activity. The enhanced catabolism of VLDL generates surface remnants, which are transferred to HDL. HDL concentrations are further augmented by an increase in PPARα - mediated transcription of apolipoprotein AI (Apo AI) and apolipoprotein AII (Apo AII). Ultimately, the rate of HDL-mediated reverse cholesterol transport may increase. Fibrates activate PPARα, which binds to a PPARα response element in conjunction with the retinoid X receptor. Other effects of fibrates include an increase in the size of LDL particles, increased removal of LDL, and a reduction in the levels of plasminogen activator inhibitor type I [37,38].

From a clinical point of view, in all available 5 randomized control trials (Table 1) the beneficial effects of major fibrates (gemfibrozil, fenofibrate, bezafibrate) were clearly demonstrated and were highly significant in patients with atherogenic dyslipidemia [39-45]. For example, fenofibrate in the FIELD study: no significance in “general population”, already significant 14% risk reduction in low HDL subgroup, 23% significant risk reduction in high triglycerides subgroup and 27% significant risk reduction in patients with atherogenic dyslipidemia [41]. In the earliest and the most successful Helsinki Heart Study with gemfibrozil, near all benefits were derived from the patients with atherogenic dyslipidemia without any impressive effects in other subgroups [43]. The same is true for bezafibrate in the BIP trial [42], for fenofibrate in the ACCORD-Lipid trial [39] and for gemfibrozil in the VA-HIT trial [44]. We can see amazing similarity among all fibrates trials. In these circumstances, the key determinant of the overall results of the trial is dependent mainly on the number of the included appropriate patients with atherogenic dyslipidemia. So, in the ACCORD-Lipid trial there were only 17% appropriate patients with atherogenic dyslipidemia (941 of 5489)! In these patients 31% risk reductions was achieved (hazard ratio = 0.69, 95% confidence interval 0.49 - 0.97, p = 0.03 for within subgroup analysis, p for interaction = 0.057). However, 83% of patients in this trial were inappropriate for a fibrate treatment. Among them, the event rate was 10.1% in both treatment groups. Thus, overall results of the ACCORD-Lipid trial did not reach significance and this inappropriate patients selection lead to fail of the study.

**Table 1:** Results from the all outcomes trials with the major fibrates (gemfibrozil, bezafibrate, fenofibrate)

**Study**	**Primary analysis: RRR (*p*value)**	**Subgroup criteria**	**Subgroup analysis:**
**(Treatment)**			**RRR (p value)**
HHS [32,43]	−34% (<0.02)	TG >200 mg/dL	−71% (0.005)
(Gemfibrozil)		LDL-C/HDL-C >5.0	
VA-HIT [44]	−22% (0.006)	Diabetes	−34% (0.004)
(Gemfibrozil)			
BIP [42]	−9.4% (0.24)	TG >200 mg/dL,	−42% (0.02)
(Bezafibrate)		HDL-C < 35 mg/dL,	
FIELD [41,45]	−11% (0.16)	TG ≥200 mg/dL	−27% (0.005)
(Fenofibrate)		HDL-C <40 mg/dL for men, <50 mg/dL for women	
ACCORD Lipid [39]	−8% (0.32)	TG ≥204 mg/dL	−31% (0.03)
(Fenofibrate)		HDL-C ≤34 mg/dL	

*RRR* – relative risk reduction.

**Figure 2:**
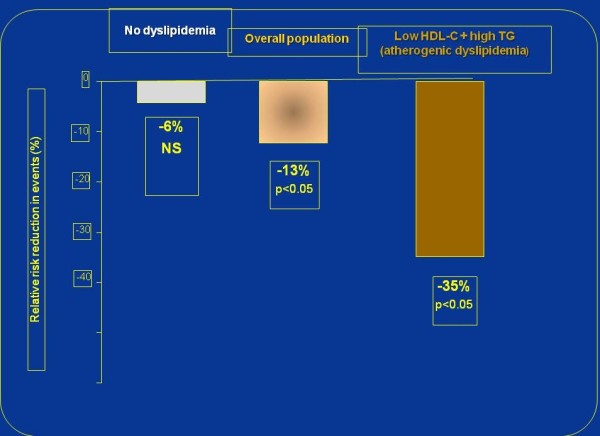
**Fibrates trials as an example of a dilution effect in epidemiology: strong significant cardiovascular risk reduction in patients with atherogenic dyslipidemia **[46]**, non significant effect in those without dyslipidemia **[46]** and a blended modest effect in the mixed “general population” **[47]**.**

In a recent meta-analysis of five dyslipidemic subgroups totaling 4726 patients, a 35% relative risk reduction in cardiovascular events was observed compared with a non significant 6% reduction in those without dyslipidemia [46]. Meta-analysis performed in a so called “general population” [47] reflecting a blend of effects in patients with and without atherogenic dyslipidemia - a “mean diluted” effect of fibrate therapy was reduced, producing only 13% RR reduction for coronary events (p < 0.0001). Figure 2 illustrates a dilution effect in epidemiology: strong significant cardiovascular risk reduction in patients with atherogenic dyslipidemia, non significant effect in those without dyslipidemia and blended modest effect in the mixed “general population”.

Therefore, in patients with atherogenic dyslipidemia (high triglycerides and low HDL-C), fibrates - either as monotherapy or combined with statins - are consistently associated with reduced risk of cardiovascular events. In patients without dyslipidemia this favorable effect - as expected - is absent.

## Fibrates: different spectrum of effects

However, different fibrates may have a somewhat dissimilar spectrum of effects. Currently only fenofibrate [39,41] was investigated in deep and proved to be effective in reducing microvascular complications of diabetes (in terms of diabetic retinopathy, progression of microalbuminuria and risk of limb amputations). However, there is no reason to suggest that other fibrates cannot do the same. The strongest hints for this were obtained in the large LEADER study when bezafibrate significantly reduced the severity of intermittent claudication for up to three years [48]. In addition, bezafibrate effectively reduced microvascular complications in a experimental study [49]. Also in the old RCTs, clofibrate was partially effective in the treatment of diabetic retinopathy due to an increased rate of absorption of hard exudates [50,51].

The underlying mechanisms of these effects are not fully elucidated. The reductions in the risk of T2DM-related retinopathy and risk of amputation with fenofibrate were apparently independent of effects on lipid parameters. The leading hypothesis included activation of PPARα which can modulate angiogenesis through a mechanism dependent on vascular endothelial growth factor [52]. Actions arising via PPARα activation are likely to be shared between all fibric acid derivatives [52]. Alternatively, influence on endothelial function, anti-inflammatory and anti-apoptotic effects and decreased circulating levels of fibrinogen could be involved [52,53].

Cardinal differences between bezafibrate and other fibrates are related to effects on glucose metabolism and insulin resistance. Bezafibrate, in contrast to other fibrates is pan - (alpha, beta, gamma) PPAR balanced activator [54,55]. Bezafibrate leads to long-term stabilization of insulin sensitivity and pancreatic beta-cell function, reduced blood glucose level and HbA1C [56-59]. In addition, bezafibrate significantly increased serum adiponectin level [60]. Multiple studies have shown that bezafibrate reduced the incidence of T2DM by 30-40% compared to placebo or other fibrates during a long-term follow-up period [61-63].

In patients with MetS, bezafibrate treatment was associated with significant 29% reduced risk of any MI and 33% reduced risk of non-fatal MI. The early decrease in MI incidence was reflected later in a tendency of reduced cardiac mortality. Of note, among patients with augmented features of MetS (4–5 risk factors for MetS) a marked 56% reduction in cardiac mortality on bezafibrate was observed [64]. Of course, caution should be used in interpreting these findings, which were identified in a post-hoc analysis.

## What is the place of fibrates in the statins world?

**Figure 3:**
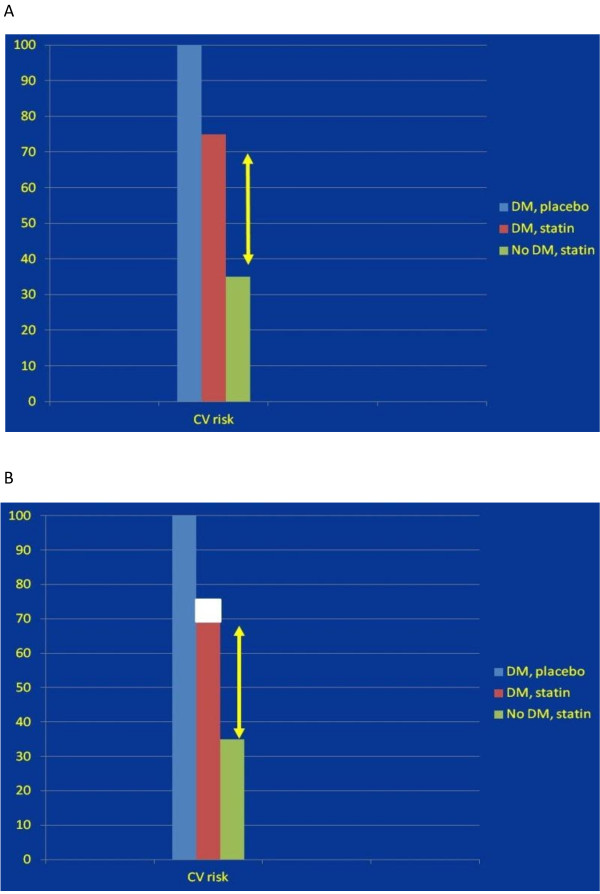
**A. Residual risk in people with diabetes and atherogenic dyslipidemia: effect of standard-dose statin therapy, pooled data **[66-74]**.** Red column represents the coronary risk in the people with diabetes and atherogenic dyslipidemia treated by statins as compared to placebo (blue column), and the patients without diabetes and atherogenic dyslipidemia treated by statins (green column). The yellow arrows demonstrate the additional coronary risk of the diabetic patients with atherogenic dyslipidemia treated by statins. **B**. Residual risk in people with diabetes and atherogenic dyslipidemia: effect of intenesive statin therapy. The hypothetical extra benefit obtained by intensive statin therapy based on the meta-analysis (it is presented by the white box) [80]. Residual risk is still remained considerable.

Until now, there were no direct “head to head” statin vs. fibrate comparisons at all. Only recently intermediate-size (274 patients) RCT have demonstrated that bezafibrate was significantly better than pravastatin (a relatively weak statin) in reduction of cardiovascular events [65]. Anyway, even *intensive statin therapy does not eliminate the cardiovascular risk associated with low HDL or/and high triglycerides (atherogenic dyslipidemia)!* Current therapeutic use of statins as monotherapy is still leaving many patients with combined dyslipidemia (which included atherogenic dyslipidemia) at high residual risk for coronary events [38,66-74]. Figure 3A is a graphic representation of the definition of residual cardiovascular risk in patients treated by conventional statin therapy. The significant residual cardiovascular risk is still present and is not affected by standard LDL - lowering therapy.

The next step in the risk reduction was the concept of “intensive” high dose statin therapy. Direct testing of varying degrees (intensive vs. conventional) of LDL-C lowering by using of active comparators (statin vs. statin) has been tested in 5 large outcomes trials [75-79]: PROVE IT--TIMI 22, A to Z, TNT, IDEAL and SEARCH. Out of the 5 trials which investigated intensive vs. standard statin regime, we have 2 “positive” with strong reservations: PROVE IT-TIMI 22 (it was based on very strange study design) and TNT (total death moved in a wrong direction) - and 3 “negative”: A to Z, IDEAL and SEARCH. Anyway, pooled data were in favour of the intensive statin therapy [80]. Figure 3B illustrates the hypothetical extra benefit obtained by intensive statin therapy based on the meta-analysis (represented by the white box). Residual risk is still remained considerable. Significant increase in side effects during intensive therapy was observed (elevations of liver enzymes, muscle aches, cognitive decline and the development of diabetes mellitus) [38,80-83].

**Figure 4:**
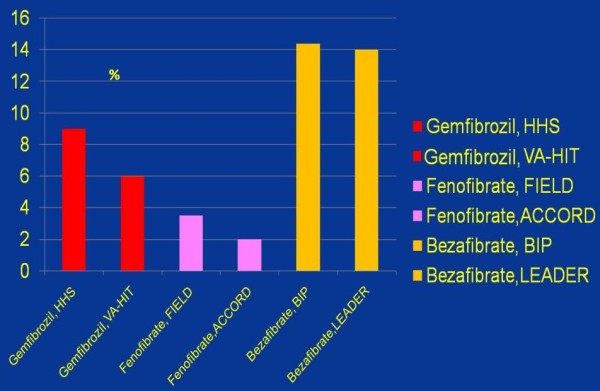
**Effect of different fibrates on HDL-C level (vs. placebo), HDL-C elevations vs. baseline during a treatment usually were significantly higher **[32]**,**[39]**,**[41-45]**,**[48].

The risk associated with high triglycerides and low HDL may be eliminated by fibrate. Among major fibrates, bezafibrate appears to have the strongest [42,48] and fenofibrate the weakest [39,45] effect on HDL-C (Figure 4). As compared with statin monotherapy (effective mainly on LDL-C levels and plaque stabilization), the association of a statin with a fibrate will also have a major impact on triglycerides, HDL and LDL particle size. Moreover, in the case of bezafibrate one could expect neutralizing of the adverse pro-diabetic effect of statins. Though muscle pain and myositis is an issue in statin/fibrate treatment, adverse interaction appears to occur to a significantly greater extent when gemfibrozil is administered. However, bezafibrate and fenofibrate seem to be safer and better tolerated [84-93].

Particularly, plasma concentration of statins are markedly increased by gemfibrozil but not by fenofibrate or bezafibrate [89,90,93]. So, gemfibrozil, which is a good “evidence-based” fibrate for monotherapy, apears to be a problematic in the “statins world”. Unfortunately, safety concerns about gemfibrozil may lead to exaggerate precautions regarding fibrate administration and therefore diminish the use of these useful agents.

In a fibrate/statin combined therapy, the statin should probably be taken at the evening and the fibrate in the morning to avoid matched peak dose concentrations. Anyway, although in clinical trials the rate of adverse events on combination was not significantly greater compared with monotherapy, clinical and laboratory monitoring of patients who receive combined treatment could be prudent.

Currently there are a few hard outcome evidences regarding a combination statin/fibrate. In ACCORD Lipid study fenofibrate leads to cardiovascular risk reduction in pre-specified subgroup of patients with atherogenic dyslipidemia [39]. In the observational study of the 150 patients, the combination of bezafibrate and simvastatin was more effective than monotherapy in reduction of cardiovascular events [92]. In the small randomized controlled trial bezafibrate on top of statin-based treatment was a safe and significantly reduced major adverse cardiovascular events (MACE) in patients with acute ST elevation MI [94]. The authors particularly emphasized in this study ability of bezafibrate significantly reduced fibrinogen levels.

Recently, new data regarding statin/fibrate combination were published using the high quality comprehensive nationwide ACSIS registry [95]. There were 8545 patients treated with statin alone and 437 patients treated with a statin/fibrate combination (mainly bezafibrate). Development of 30-day MACE (primary end-point) was recorded in 6.0% patients from the statin monotherapy group vs. 3.2% from the combination group, (p = 0.01). 30-day re-hospitalization rate was also significantly lower in the combination group. Kaplan-Meier analysis of total mortality during one year was close to significance in favor of the combination (p = 0.066). Multivariate analysis identified the fibrate/statin combination as an independent predictor of 46% reduced risk of MACE in overall population (p = 0.03). In the subgroup interaction analysis the most impressive results were found in the subgroup with diabetes and atherogenic dyslipidemia. As one could expect, in patients without dyslipidemia this effect was absent. It should be emphasized that even though these “real world” observation data cannot replace RCT, the consistency of the results supports their credibility.

## Conclusions

Even intensive statin therapy does not eliminate the residual cardiovascular risk associated with atherogenic dyslipidemia (low HDL and high triglycerides). Meta-analyses of randomized control trials clearly demonstrated that the main fibrates significantly reduce this risk. Combined fibrate/statin therapy is more effective in achieving a comprehensive lipid control and may lead to additional cardiovascular risk reduction, as could be suggested for fenofibrate following ACCORD Lipid study subgroup analysis and for bezafibrate on the bais on one small randomized study and multiple observational data. Therefore, in appropriate patients with atherogenic dyslipidemia fibrates- either as monotherapy or combined with statins – are consistently associated with reduced risk of cardiovascular events. Fibrates currently are an indispensable part of the modern anti-dyslipidemic arsenal for patients with atherogenic dyslipidemia.

## Abbreviations

ACSIS: Acute Coronary Syndromes-Israel Survey; BIP: Bezafibrate Infarction Prevention study; CETP: Cholesteryl ester transfer protein; CHD: Coronary heart disease; FA: Fatty acids; HDL-C: High density lipoprotein cholesterol; LDL-C: Low density lipoprotein cholesterol; LPL: Lipoprotein lipase; MACE: Major adverse cardiovascular events; MI: Myocardial infarction; PPARs: Peroxisome proliferators-activated receptors; RCT: The randomized control trials; VLDL: Very-low-density lipoprotein.

## Competing interests

AT received speaker fee and travel expenses support from Abbott, Tribute, Novartis and Merck. EZF declares that he has no competing interests.

## Authors’ contribution

Both authors have equally contributed in the conception and drafting of the manuscript. Both authors read and approved the final manuscript.
